# How intrinsically motivating are swimming instructors/lessons in the Netherlands? An observational study through the lens of self-determination theory

**DOI:** 10.3389/fspor.2023.1236256

**Published:** 2023-09-20

**Authors:** Carola Minkels, John van der Kamp, Peter J. Beek

**Affiliations:** ^1^ InnoSportLab de Tongelreep, Eindhoven, Netherlands; ^2^ Department of Human Movement Sciences, Amsterdam Movement Sciences, Vrije Universiteit Amsterdam, Amsterdam, Netherlands

**Keywords:** intrinsic motivation, self-determination theory, swimming, water safety, instructors, basic psychological needs

## Abstract

**Background:**

It is desirable that (more) children continue swimming after having completed their swimming lessons to preserve their swimming skills and water safety, and as part of an active, healthy lifestyle. This may be encouraged by stirring children's intrinsic motivation for swimming during swimming lessons. However, it is currently unknown how intrinsically motivating swimming lessons are in Western countries.

**Purpose:**

This study examined to what extent swimming instructors in the Netherlands cater to the basic needs of autonomy, competence, and relatedness, which, according to Self-Determination Theory (SDT), promote intrinsic motivation. Additionally, it examined whether an SDT-based teaching program prompts instructors to better meet these needs, and to what extent the teaching program, the education and experience of the instructor, and the group size predict the employment of SDT in swimming lessons.

**Methods:**

A total of 128 swimming lessons given by equally many instructors were observed in the Netherlands and rated on a modified version of the SDT teaching style scale to assess autonomy, competence, and relatedness support. The swimming lessons referred to four teaching programs, one of which was explicitly based on SDT.

**Results:**

Instructors exhibited autonomy-thwarting, weakly competence-supportive, and relatedness-supportive behaviors. The SDT-based teaching program scored higher on the provision of autonomy, competence, and relatedness in lessons. This finding was significant for autonomy. Teaching program was the only significant predictor of SDT employment by instructors.

**Conclusion:**

Further improvement is desirable in catering to the basic needs, particularly autonomy, which can be achieved by deliberately implementing the principles of SDT into teaching programs for swimming.

## 1. Introduction

All over the world children receive swimming lessons to secure their safety in water (1). In Western countries, most children are introduced to swimming in this manner, either through swimming schools and clubs, or privately by their parents or guardians. Consequently, three out of four people are able to swim (2). Among the frontrunners are Australia, Canada, and Northwestern Europe, where approximately 85% of adult women and 90% of adult men can swim (2). Nevertheless, the percentage of children who join a swim sports club after having acquired basic swimming skills is low. For example, in the United States of America, only 0.34% of children under 15 are a member of a swimming club indicating that, among the organized sports, swimming is far less popular than, for instance, basketball, soccer, and ice hockey (3–5). This may have several causes, including the fact that swimming can be a very expensive sport, especially as the competition level increases (6). Similar trends have been reported for the Netherlands (7, 8). This raises the question how to stimulate more children to practice swimming as a sport, or to continue swimming in unorganized form, and how swimming lessons may be of help in this regard.

Before addressing this question, it is useful to highlight the benefits of actively continuing to swim after having learned to swim. Swimming is a very healthy, whole body physical activity that requires a mix of endurance and strength (9). Additionally, it has low injury prevalence and can be practiced well into old age (10). Moreover, experienced swimmers have a lower risk of drowning than inexperienced swimmers, provided that swimming skills are maintained (11). Research has shown that of the 80% of Americans who acquired the ability to swim, only 56% can perform the five basic skills that are required to survive in water (i.e., tread water for 1 min without using a floating device, step into water over the head and return to the surface, turn around in a circle in the water and find an exit, swim 25 yards without stopping, exit a pool not using a ladder) sufficiently well. In all likelihood, this is due to a gradual decline in swimming skills (12). Regularly recurring practice is necessary to preserve swimming skills, and to master swimming in more challenging environments, such as strong currents and waves (11). Since most children in Western countries acquire swimming skills during swimming lessons, these lessons also offer an ideal opportunity to stimulate children to adopt swimming as a sporting activity, be it at a swimming club or in unorganized form. To this end, swimming lessons should be focused not only on the acquisition of swimming skills to become water safe, but also on developing motivation for and pleasure in swimming.

The latter aim may be achieved by incorporating the tenets of the self-determination theory (SDT) of Ryan and Deci (13) into swimming lessons. SDT is a motivation theory that distinguishes distinct motivating factors when performing an activity (14). The most basic distinction in SDT, as indeed all theories of motivation, is that between intrinsic and extrinsic motivation (14). Intrinsic motivation refers to performing activities out of mere interest, enjoyment, and/or satisfaction in that activity, whereas extrinsic motivation points to performing activities to avoid punishment or guilt, to obtain approval or rewards, or to support personally held values (14). It is assumed that the acquisition and regularly recurring practice of a novel skill is facilitated when the individual is intrinsically motivated to engage in the activity in question (14, 15). According to SDT, three basic psychological needs are important in stimulating the development of intrinsic motivation: autonomy, competence, and relatedness (13). Autonomy involves feelings of willingness and choice concerning activities undertaken (13). Children's perception of autonomy can be strengthened by creating opportunities for initiative and by providing choices and rationales to children (16, 17). Competence refers to perceptions of being able to master challenges (13). This is achieved best within well-structured environments that safely offer challenges, with positive feedback, and opportunities to grow (16). Lastly, relatedness entails a sense of belonging and connection (e.g., with teachers, peers, siblings, and parents) (13). Perceptions of relatedness can be increased by promoting interaction between children, letting children practice and play together, being kind and warm to children, and taking children's feelings into account (16).

In educational settings, the extent to which autonomy, competence, and relatedness are incorporated into an activity is reflected in a teacher's teaching style. Teaching style refers to the overall education and management strategies employed by teachers and has been classified as either autonomy- or control-oriented (18). In an autonomy-oriented teaching style, teachers try to identify, nurture, and develop children's intrinsic motivational resources (19, 20). This is the opposite of a control-oriented teaching style in which teachers try to pressure children to think, feel, or behave in a specific way (19, 20). According to SDT, it is likely that children become intrinsically motivated to perform the taught activities when teachers employ an autonomy-oriented teaching style, in which the three basic psychological needs of SDT are integrated in those activities (15, 21). Conversely, thwarting any of the three basic needs by employing a control-oriented teaching style will likely lead to a decrease in intrinsic motivation (15, 21). Through the lens of SDT, the question is therefore to what extent swimming instructors exhibit an autonomy-oriented teaching style and cater to its three basic needs during contemporary swimming lessons. After all, swimming instructors play an important role in the development of swimming skills in children, and thus in stirring their intrinsic motivation for swimming. To date, however, the teaching style of swimming instructors has not been a subject of research. We therefore deemed it worthwhile to conduct a study in a representative Western country, i.e., the Netherlands, to gain more insight into this important topic.

Furthermore, considering that the teaching style of swimming instructors may be associated with relatively stable personality traits, it is of interest to examine whether it is possible to make an individual swimming instructor's teaching style more autonomy-oriented, if deemed necessary or desirable. Reeve (22) examined this question in an academic context by training preservice teachers (other than physical education teachers) in autonomy-supportive, controlling, or neutral instructional strategies. The results showed that teachers exposed to autonomy-supportive instructional training were more autonomy-supportive after training than teachers exposed to neutral or controlling instructional training, thus indicating that the autonomy-oriented teaching style is teachable. However, Reeve (22) assessed teaching style using self-reported questionnaires, which are not capable of proving that also the actual in-class teaching style was changed. This aspect was investigated by Tessier, Sarrazin, and Ntoumanis (23) in a similar study with physical education teachers. Trained raters assessed the actual in-class provision of autonomy, competence, and relatedness by physical education teachers before and after they had been taught about the need for SDT-based teaching behaviors in class. The raters completed an adapted version of the 7-point Likert observation scale developed earlier by Reeve, Jang et al. (16); (i.e., the SDT teaching style scale). Results showed that, after the SDT-based intervention, teachers displayed significantly more in-class behaviors that cater to autonomy, competence, and relatedness than before the intervention. Tessier et al. (23) thus concluded that teachers can learn to better cater to the three basic psychological needs in class [see also (16, 24–31)]. It is important to note in this context that while the internal validity of the SDT teaching style scale has been confirmed (16), its structural validity remains to be established. This can be achieved by means of a confirmatory factor analysis, which should reveal that autonomy, competence, and relatedness are indeed separate factors.

The question is whether the finding that teaching style is malleable generalizes to swimming instructors, which might not be the case for several reasons. Swimming lessons, school classes, and physical education lessons are all pedagogical situations where teachers try to teach children new skills. However, there are relevant differences between these learning environments. First, compared to regular school classes and also most physical education lessons, it is critically important to secure children's safety in the water during swimming lessons. Consequently, it may be more difficult to apply an autonomy-oriented teaching style during swimming lessons as this may endanger children's safety. Second, children in swimming lessons are typically aged between 5 and 7 years old, which is much younger than the students in the studies of Reeve (22) and Tessier et al. (23). Teachers may perceive children at that age as too young or immature to respond adequately when they are given (more) autonomy. Hence, it remains to be determined whether the findings of Reeve (22) and Tessier et al. (23) also apply to swimming instructors, and to what extent they can be taught and are inclined to better cater to children's needs for autonomy, competence, and relatedness.

In the Netherlands, three basic levels of swimming competence are distinguished, referred to as A, B, and C. Each level consists of a strictly defined set of skills and criteria. Once the skills corresponding to a certain level have been acquired, children can obtain a diploma by demonstrating those skills in an exam judged by a licensed swimming instructor. Although the skills that need to be demonstrated are strictly defined, there are no regulations regarding the way they should be taught in swimming lessons. As a result, considerable variation exists in the pedagogical and didactical principles and assumptions underpinning swim teaching programs in the Netherlands. Several organizations have developed such teaching programs, among which Easyswim, ZwemABC (NRZ), Optisport, and SuperSpetters (KNZB) are the most prominent. These programs have distinct objectives. Inspired by SDT, Easyswim strives to deliver engaging swimming lessons that are tailored to the specific needs of children (32). ZwemABC aims to secure the safety of individuals in and around water by teaching children the lifesaving skills delineated by the A, B, and C proficiency levels (33). Optisport strives to facilitate the acquisition of swimming skills in children by capitalizing on their imagination and providing a fun and engaging learning environment (34). Lastly, SuperSpetters aims to teach children to swim with a proper swimming technique (35). Of these teaching programs, Easyswim is the only one that explicitly incorporates the principles of SDT into their teaching program and systematically trains swimming instructors in strengthening children's needs for autonomy, competence, and relatedness. It differs from the other programs by aiming to have the children practice relatively independently of the instructor. To gain insight into the extent to which swimming instructors can be taught to apply the principles of SDT and exhibit more autonomy-oriented teaching styles, we examined whether swimming instructors associated with Easyswim better cater to the basic needs in comparison with swimming instructors associated with other swimming programs.

Furthermore, in the Netherlands, there are three different ways to become a swimming instructor (general PE teacher education; general swimming instructor education; and swimming instructor education tailored to a specific program). These swimming instructor education programs all differ in the extent to which they address the SDT, if at all. Therefore, we also examined whether the education background influences the extent to which swimming instructors cater to the three basic needs during swimming lessons.

Finally, there may be several other factors that affect the employment of the principles of SDT in swimming lessons. For example, in educational research, Leroy, Bressoux, Sarrazin, and Trouilloud (36) found that experienced teachers established more intrinsically motivating classroom climates and better supported their students' psychological needs in comparison to inexperienced teachers. Additionally, Good, Grouws, Mason, Slavings, and Cramer (37) suggested that group size affected the employment of SDT in class. Smaller group sizes resulted in better content attunement to children's ability levels, presumably catering to their perceived competence (37). Smaller group sizes also promoted self-management, student interaction, and student cooperation (37), bolstering autonomy and relatedness, respectively. However, since this research was done in academic activities in schools, it is unclear whether experience and group size also affect the teaching style of swimming instructors.

The aims of the present study were threefold. The primary aim was to examine to what extent swimming instructors in the Netherlands cater to the three basic psychological needs during swimming lessons. The second aim was to examine whether a teaching program based on the principles of SDT prompts swimming instructors to better meet the three basic psychological needs in comparison with other teaching programs. The third aim was to examine to what extent the applied teaching program, the instructor's education and experience, and the group size predict the employment of SDT principles in swimming lessons. To accomplish those aims, we first had to confirm the validity and reliability of Tessier et al.'s (23) SDT teaching style scale that we modified to assess the swimming instructors' teaching styles.

Regarding the first aim, we based our expectation on the commonly held but untested opinion about the state of swimming lessons in the Netherlands, namely that swimming instructors typically tend toward need-thwarting behaviors. Based on this general opinion, we expected to find average scores on the three subscales for autonomy, competence, and relatedness below a neutral 4. Regarding the second aim, we expected that the swimming program based on the principles of the SDT would prompt swimming instructors to better meet the three basic psychological needs of autonomy, competence, and relatedness, considering that previous research in other domains has shown that it is possible to change a person's teaching style to become more autonomy-oriented (16, 22, 23, 29, 31). Concerning the third aim, we expected that the swimming program, the swimming instructor's education and experience, and the number of children in swimming lessons would predict the degree of catering to the three basic psychological needs, considering that in other educational domains these were demonstrated to affect the degree to which teachers showed an autonomy-oriented teaching style (23, 36, 37).

## 2. Methods

### 2.1. Participants

A power analysis (G*Power, 3.1.9.6, Kiel, Germany) for a one-way omnibus ANOVA with fixed effects was conducted to determine the number of swimming instructors (and thus the number of swimming lessons) that had to be observed for the study to be statistically meaningful. The power analysis with a moderate effect size f of 0.30, an alpha error probability of 0.05, and a power of 0.8, revealed that a minimum sample size of 128 swimming instructors providing an equal number of swimming lessons was required.

Swimming instructors in the Netherlands were invited to participate in the study through the nation-wide professional networks of the Nationale Raad Zwemveiligheid (NRZ) and the national swimming center, InnoSportlab de Tongelreep at Eindhoven. Through these networks, the required sample size of 128 swimming instructors was achieved (age = 40.28 ± 14.51 years; 101 female, 27 male; mean ± standard deviation), who were affiliated to a total of 42 different swimming schools. The 128 observed swimming lessons were distributed as follows over the four teaching programs: Easyswim 32; ZwemABC 31; Optisport 33; and SuperSpetters 32. The swimming lessons were observed for the entire duration to fill out the SDT teaching style scale (see below). The lessons involved novice swimmers between 4 and 10 years of age, who were taught the leg movements of the different swimming strokes.

Ethical clearance of the study was provided by the Ethical Committee of the Faculty of Behavioral and Movement Sciences of the Vrije Universiteit Amsterdam (VCWE-2022-075) and all swimming instructors provided written informed consent before participation. Because the study concerned the observation of a regular educational activity, and involved no interventions nor the collection of data from individually identifiable children, it was sufficient to ask the instructors for permission and to inform the parents.

### 2.2. Apparatus and equipment

All verbalizations of the swimming instructors were recorded on audiotape using a waterproof microphone (Instamic Pro) when the swimming instructor taught from the water and a non-waterproof microphone (Debra DVO2) when the swimming instructor taught from the poolside. No video recordings were made in view of privacy considerations.

During the swimming lessons, practice aids such as kickboards, surfboards, fins, and trunks were provided to the children. Materials to climb over, jump over, swim through, and pick up from the pool floor were also used. No constraints were imposed on the use of swimming aids and materials.

To assess the extent of autonomy, competence, and relatedness support exhibited in the swimming lessons, the modified SDT teaching style scale of Tessier et al. (23) was used and adapted for the purpose of the present study (Supplementary Material I). This scale was chosen in view of its prior utilization in PE settings (23). The scale consists of three different subscales which measure the teacher's provision of autonomy support (autonomy), the teacher's provision of structure (competence), and the teacher's provision of involvement (relatedness), respectively. Concerning the subscale autonomy, we merged the four original items (organizational instructions given to the whole class, rationales given to the whole class, organizational instructions during teacher-student interaction, rationales during teacher-student interaction) into two general items (organizational instructions and rationales). This was done in view of the much smaller group sizes in swimming lessons (i.e., between 5 and 10 children) compared to physical education classes in previous studies (i.e., between 20 and 30 students). Furthermore, given that the motivational strategies and language used by teachers can also impact children's perceived feelings of autonomy support, two items measuring these constructs from the scale of Reeve, Jang et al. (16) were added to the subscale autonomy. The subscale competence was left unaltered. One extra item was added to the subscale relatedness to evaluate the interaction between children. As posited by Ryan and Deci (13), relatedness entails a sense of belonging not only with one's teacher but also with one's peers. Additionally, Reeve, Jang et al. (16) stated that perceptions of relatedness can be increased by promoting interaction between children and letting children practice and play together. Hence, it was deemed theoretically meaningful to include an additional item assessing the interaction between children. Each item was scored on a 1–7 Likert scale with 1 indicating “behaviors that thwart the basic psychological need” and 7 indicating “behaviors that nurture the basic psychological need”. Tessier et al. (23) took the scale's midpoint (i.e., 4) as a threshold to distinguish between need-supportive and need-thwarting behaviors in which 1 to 3 points indicate need-thwarting behavior, 4 points need-neutral behavior, and 5 to 7 points need-supportive behavior. Both the swimming instructor's verbal and non-verbal behaviors were considered. Furthermore, Reeve, Jang et al. (16) provided evidence for the internal validity of the scale, while Tessier et al. (23) showed that the scale is reliable (α_autonomy_ = 0.70, α_competence _= 0.83, α_relatedness _= 0.84, ICC_total_ = 0.80). A pilot test of the modified SDT teaching style scale was performed in which two instructors were observed by four independent raters. From the resulting pilot data, the inter-rater reliability was calculated using intraclass correlation coefficient (ICC) based on a mean-rating (k = 2), absolute-agreement, Two-Way Mixed-Effects Model. This yielded the following results: ICC_total_ = 0.905, 95% CI [0.813, 0.958], *p* < 0.01; ICC_autonomy_ = 0.935, 95% CI [0.813, 0.985], *p* < 0.01; ICC_competence_ = 0.857, 95% CI [0.315, 0.996], *p* < 0.01; ICC_relatedness_ = 0.974, 95% CI [0.911, 0.996], *p* < 0.01, indicating good to excellent reliability (38). The pilot-test observation data were not included in the analysis of the observation data proper.

A background information questionnaire was developed to obtain information about which education program instructors had followed to become a swimming instructor, how many years and how many hours per week instructors provided swimming lessons, and the number of children in the swimming lesson (Supplementary Material II).

### 2.3. Procedure and design

Students volunteered to observe the lessons provided by the swimming instructors as part of their education as human movement scientists. They were educated about the SDT and subsequently trained how to apply the modified SDT teaching style scale during a two-hour meeting led by the first author (CM). After the meeting, the students observed two swimming lessons and completed the modified SDT teaching style scale. Analysis of their scores revealed that sufficient reliability among raters had been achieved (see above).

Before observing the swimming lesson, the swimming instructor was informed about the observation procedure and given the opportunity to ask questions for clarification. We told the swimming instructor that the aim of the observation was to gain insight into the current teaching programs used during swimming lessons in the Netherlands. To this end, we asked the swimming instructor to provide a swimming lesson as usual. Specific aspects of the study, such as the exact aims of the study and the items observed, were not disclosed to the swimming instructor to avoid conscious adaptions of the swimming lesson or teaching style by the swimming instructor. Only after the observations were performed, the aims of the study were fully disclosed to the swimming instructor.

Every swimming lesson was observed independently by two trained raters, who were seated at separate locations but close to the pool to obtain an encompassing view of the swimming lesson. The modified SDT teaching style scale was completed at the end of the swimming lesson. In case of doubt, or if the observation was hindered because of poor acoustics, the raters listened to the audio recordings afterward and then completed the modified SDT teaching style scale. In cases where the raters' assessments did not align, the average score was computed and used in subsequent analyses.

When the swimming lesson was finished, the background information questionnaire was administered.

### 2.4. Data processing

The data were prepared for statistical analysis in R studio (Rstudio-2022.07.2-576).

#### 2.4.1. SDT scores

The total score on the modified SDT teaching style scale was calculated by averaging the ratings of all items, and then averaging it over the two raters. Additionally, the average scores for the items of the subscales autonomy, competence, and relatedness were calculated and also averaged over the two raters. Since the data were ordinal, the median instead of the mean was calculated for the total and subscale scores.

#### 2.4.2. Instructor's education

Supplementary Material III contains an overview of instructor's education programs accepted by the NRZ. In accordance with this list, the percentage of certified swimming instructors was calculated. Supplementary Material IV contains a list of the swimming instructor's education programs that were included in the analysis. We classified the education programs into three distinct categories: general PE teacher education, general swimming instructor education, and swimming instructor education tailored to a specific program.

#### 2.4.3. Instructor's experience

The instructor's experience was determined by multiplying the number of hours they taught per week by 44 (i.e., the number of weeks in a year minus holiday weeks), and then multiplying the resulting product by the number of years they had been teaching swimming lessons.

#### 2.4.4. Group size

During each swimming lesson, one of the raters counted and recorded the number of children in the observed swimming lessons.

### 2.5. Statistical analyses

All statistical procedures were performed in Rstudio (Rstudio-2022.07.2-576). Statistical significance was set at *p* < 0.05.

#### 2.5.1. Structural validity of modified SDT teaching style scale

Confirmatory factor analysis was performed to check the structural validity of the modified SDT teaching style scale. The diagonally weighted least squares (DWLS) method was used for this analysis in view of the ordinal character of the data. It was tested whether the data fitted the hypothesized correlated three-factor model of the modified SDT teaching style scale (i.e., that the scale contains autonomy, competence, and relatedness as subscales). Items of the specific factors were constrained to load on the factor they belonged to (Supplementary Material I).

Chi-square statistic (*X*^2^), Comparative Fit Index (CFI), Tucker-Lewis Index (TLI), Root Mean Square Error of Approximation (RMSEA), and Standardized Root Mean Square Residual (SRMR) were used as model fit tests (39). Non-significant *p*-values of the Chi-square statistic indicated a good fit (40). Furthermore, for the CFI and the TLI, values above 0.90 indicated an acceptable fit (40, 41). Lastly, for the RMSEA and SRMR, values below 0.08 indicated an acceptable to good fit (40, 41).

#### 2.5.2. Reliability of modified SDT teaching style scale

Inter-rater reliability was assessed for the total SDT score, as well as for the scores on the subscales autonomy, competence, and relatedness. To this end, ICC based on a mean-rating (k = 2), absolute-agreement, One-Way Random-Effects Model was used. ICC values above 0.75 indicated good reliability (38). The internal consistencies of the total and subscores were determined using Cronbach's alpha with values of 0.70 or higher indicating sufficient reliability (42). Furthermore, standard error of measurement (SEM), minimal detectable change at the group level (MDC_group_), and minimal detectable change at the individual level (MDC_individual_) were assessed (43, 44).

#### 2.5.3. Relation of teaching program, instructor's education and experience, and group size to instructor's employment of SDT

Since the scale's midpoint (i.e., 4) was the threshold to distinguish between need-supportive and need-thwarting behaviors (23), we conducted One-Sample Wilcoxon tests Signed Rank Tests to determine whether the median of our sample was equivalent to 4. Furthermore, considering that the collected data were on an ordinal scale, a Kruskal-Wallis test was performed to examine whether the subscores on the SDT teaching style scale differed for the three basic needs (i.e., autonomy, competence, relatedness).

Kruskal-Wallis tests were performed to determine the effect of teaching program (group: Easyswim, ZwemABC, Optisport, SuperSpetters) on instructor's employment of SDT, instructor's autonomy support, instructor's provision of competence, and instructor's provision of relatedness in swimming lessons. Post hoc analyses were conducted using Wilcoxon's rank sum test with a Bonferroni correction of the alpha level to *p* = 0.01.

Additionally, a hierarchical four-stepped linear regression analysis was performed to examine whether the teaching program (i.e., Easyswim, ZwemABC, Optisport, and SuperSpetters), the instructor's education and experience, and the group size predicted total and subscores on the modified SDT teaching style scale. Teaching program was entered as the first step in the regression, experience as the second step, education as the third step, and group size as the last step. Relevant assumptions were tested before conducting the hierarchical regression analysis. Q-Q plot analyses revealed that the data were normally distributed. Inspections of the residual plots showed that the assumptions of homogeneity of variance and linearity were met. Lastly, VIF values around 1 revealed that the assumption of multicollinearity was also met.

## 3. Results

### 3.1. Descriptive statistics

All 128 swimming instructors were included in the analysis. Table 1 shows the characteristics of the swimming instructors for each teaching program. The experience of the swimming instructors ranged from 2 months to 50 years with an average of 13.90 ± 11.98 years. Most instructors (86.7%) were certified to provide swimming lessons, group sizes ranged from 2 to 21 children (8.23 ± 2.99), and the swimming lessons lasted between 30 and 120 min (48.67 ± 15.25).

**Table 1:** Swimming Instructors’ characteristics for each teaching program (mean ± standard deviation).

	Easyswim (*n* = 32)	ZwemABC (*n* = 31)	Optisport (*n* = 33)	SuperSpetters (*n* = 32)
Age (years)	36.24 ± 12.18	44.52 ± 13.31	43.55 ± 14.81	37.21 ± 16.14
Gender (percentage female)	68.75%	90.32%	78.79%	78.13%
Experience (years)	9.40 ± 10.90	17.15 ± 11.99	16.80 ± 11.78	12.26 ± 12.00
Education (percentage certified)	93.75%	87.10%	93.94%	71.88%
Group size (number of children)	7.16 ± 2.05	8.55 ± 3.38	9.79 ± 3.35	7.41 ± 2.30
Duration swimming lesson (minutes)	48.75 ± 13.56	46.45 ± 11.85	50.91 ± 14.81	48.44 ± 19.90

### 3.2. Structural validity of the modified SDT teaching style scale

Model fit indices showed acceptable to good fit for CFI, TLI, and SRMR (CFI = 0.988; TLI = 0.983; SRMR = 0.078), and poor fit for *X*^2^ and RMSEA (*X*^2^(32) = 64.920, *p* = 0.001; RMSEA = 0.09, 90% CI [0.058, 0.121]). These results are collated in Table 2. Standardized item-factor loadings were all in the expected direction (i.e., positive) and statistically significant. Additionally, most item-factor loadings were substantial (>0.4) (45). Item C1 (degree of differentiation) and item R3 (interaction between children) demonstrated weak item-factor loadings of 0.23 and 0.32, respectively (45). It was, therefore, assumed that improvements in model fit could be gained by removing these two items. Following the elimination of item C1, the model fit indices indicated acceptable to good fit for CFI, TLI, and SRMR and poor fit for *X*^2^ and RMSEA (Table 2). After removal of item R3, and subsequently of both items R3 and C1, all model fit indices became acceptable to good as shown in Table 2. Ultimately, the decision was made to exclude only item R3 from the analysis. Although item R3 was deemed theoretically meaningful for evaluating the relatedness among children during a swimming lesson, its removal rendered all model fit indices acceptable, thereby resulting in an adequate model permitting a valid and trustworthy interpretation of the results obtained.

**Table 2:** Confirmatory factor analysis of the modified SDT teaching style scale.

Factor structure	*X* ^2^	df	*p*-value (*X*^2^)	CFI	TLI	RMSEA (90% CI)	SRMR
All items included	64.920	32	0.001	0.988	0.983	0.090 (0.058–0.121)	0.078
Exclusion of item C1	51.192	24	0.001	0.990	0.985	0.094 (0.058–0.130)	0.077
Exclusion of item R3	34.560	24	0.075	0.996	0.994	0.059 (0.000–0.100)	0.062
Exclusion of items C1 and R3	22.339	17	0.172	0.998	0.997	0.050 (0.000–0.100)	0.056

Item C1, degree of differentiation; Item R3, interaction between children; *X*^2^, chi-square statistic; df, degrees of freedom; CFI, comparative fit index; TLI, tucker-lewis index; RMSEA, root mean square error of approximation; SRMR, standardized root mean square residual.

### 3.3. Reliability of the modified SDT teaching style scale

Table 3 summarizes the reliability characteristics of the modified SDT teaching style scale. It presents data on the total score and subscores for the three psychological needs, both with and without item R3 included. Throughout the remainder of the article, all results are discussed based on analyses conducted after exclusion of item R3 from the SDT teaching style scale. The scale's interrater reliability was found to be good to excellent (ICC_total_ = 0.94, 95% CI [0.93, 0.94], *p* < 0.01; ICC_autonomy_ = 0.96, 95% CI [0.95, 0.96], *p* < 0.01; ICC_competence_ = 0.85, 95% CI [0.83, 0.86], *p* < 0.01; ICC_relatedness_ = 0.86, 95% CI [0.84, 0.87], *p* < 0.01). The scale's internal consistency for autonomy was somewhat below the threshold of 0.70 showing a Cronbach's alpha value of 0.65 [95% CI (0.55, 0.76)]. The relatedness subscale demonstrated sufficient internal consistency (α = 0.91, 95% CI [0.87, 0.94]). The subscale competence demonstrated poor internal consistency as reflected by a Cronbach's alpha value of 0.53 [95% CI (0.39, 0.68)]. Removal of item C1 (degree of differentiation) would have resulted in an improved Cronbach's alpha value of 0.67. However, we decided not to remove item C1, because it was part of the original scale developed by Tessier et al. (23), which had a Cronbach's alpha value of 0.83. The standard error of measurement for the total scale was 0.40, the minimal detectable change for the group as a whole was 0.16, and the minimal detectable change at the individual level was 1.11.

**Table 3:** Reliability measures.

	All items included	Exclusion of item R3
ICC (95% CI)		
Total scale	0.94 (0.93–0.95)	0.94 (0.93–0.94)
Autonomy	0.96 (0.95–0.96)	0.96 (0.95–0.96)
Competence	0.85 (0.83–0.86)	0.85 (0.83–0.86)
Relatedness	0.95 (0.95–0.96)	0.86 (0.84–0.87)
Internal consistency (alpha) (95% CI)		
Total scale	0.81 (0.75–0.85)	0.81 (0.76–0.86)
Autonomy	0.65 (0.55–0.76)	0.65 (0.55–0.76)
Competence	0.53 (0.39–0.68)	0.53 (0.39–0.68)
Relatedness	0.68 (0.58–0.78)	0.91 (0.87–0.94)
SEM		
Total scale	0.40	0.40
Autonomy	0.41	0.41
Competence	0.53	0.53
Relatedness	0.34	0.20
MDC_group_		
Total scale	0.16	0.16
Autonomy	0.20	0.20
Competence	0.28	0.28
Relatedness	0.18	0.11
MDC_individual_		
Total scale	1.11	1.11
Autonomy	1.14	1.14
Competence	1.47	1.47
Relatedness	0.93	0.57

ICC, intraclass correlation coefficient; MDC, minimal detectable change; SEM, standard error of the measurement.

### 3.4. Employment of SDT

**Figure 1:**
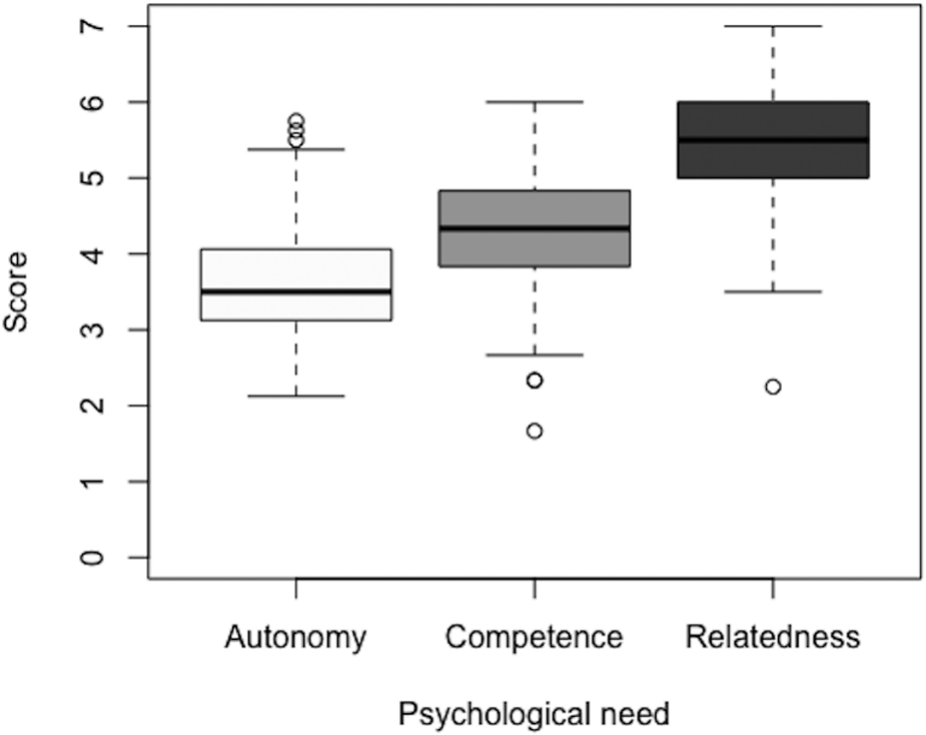
Scores for the three basic psychological needs. The bold horizontal black lines indicate the median, the grey areas the interquartile range, and the vertical dotted lines the range of the scores. The circles represent outliers.

The median score for the total scale was 4.28 points, while the median scores on the subscales autonomy, competence, and relatedness were 3.50, 4.33, and 5.50 points, respectively (Figure 1). The median subscore for autonomy was significantly lower than 4 (V = 1,823, *p* < 0.01), while those for the total score (V = 5,671, *p* < 0.01), competence (V = 5,144, *p* < 0.01), and relatedness (V = 7,285.5, *p* < 0.01) were significantly higher than 4. Additionally, the Kruskal-Wallis test showed a significant effect of need on subscores on the SDT teaching style scale, H(2) = 189.24, *p* < 0.01. Post hoc analyses with Bonferroni correction revealed that instructors scored significantly lower on the employment of autonomy in swimming lessons than on the employment of competence (W = 4,004, *p* < 0.01) and relatedness (W = 984.5, *p* < 0.01). Additionally, relatedness was better employed in swimming lessons than competence (W = 2,544.5, *p* < 0.01).

**Figure 2:**
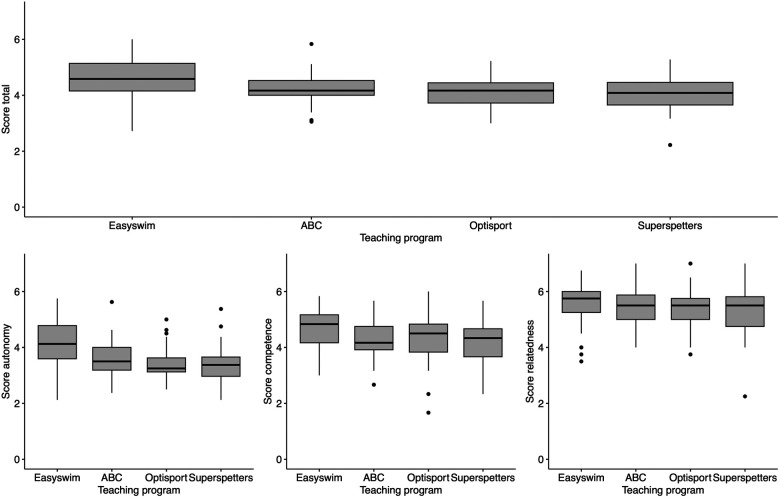
Total (top) and subscores (bottom) for the three basic psychological needs according to teaching program. The bold horizontal black lines indicate the median, the grey areas the interquartile range, and the vertical lines the range of the scores. The circles represent outliers.

Figure 2 provides a summary of the results on the three basic psychological needs for each teaching program. The Kruskal-Wallis test showed a significant effect of teaching program on the total score of the modified SDT teaching style scale, H(3) = 11.666, *p* < 0.01. Post hoc analyses with Bonferroni correction revealed that swimming instructors associated with Easyswim had significantly higher total scores than swimming instructors affiliated with Optisport (W = 742, *p* < 0.01) and SuperSpetters (W = 728.5, *p* < 0.01). Swimming instructors associated with Easyswim also demonstrated higher total scores than swimming instructors associated with ZwemABC. However, this difference was not significant after Bonferroni correction (W = 672.5, *p* = 0.02).

The Kruskal-Wallis test also revealed a significant effect of teaching program on the subscore for autonomy, H(3) = 20.192, *p* < 0.01. Post hoc analyses with Bonferroni correction demonstrated that swimming instructors affiliated with Easyswim had significantly higher autonomy subscores than swimming instructors associated with ZwemABC (W = 693, *p* = 0.007), Optisport (W = 820, *p* < 0.01), and SuperSpetters (W = 789.5, *p* < 0.01). Figure 2 suggests that instructors affiliated with Easyswim also scored higher on the provision of competence and relatedness, however, Kruskal-Wallis tests showed no significant differences between teaching program for competence [H(3) = 7.52, *p* = 0.057] and relatedness [H(3) = 1.64, *p* = 0.65].

### 3.5. Hierarchical multiple regression

The hierarchical multiple regression revealed that in step one, teaching program contributed significantly to the regression model, F(3, 124) = 4.87, *p* < 0.01, and accounted for 10.5% of the variation in the total SDT score. Adding the instructor's experience, education, and group size in subsequent steps did not result in a significant increase in R^2^ (Table 4). Also with regard to the subscore for autonomy, the hierarchical multiple regression revealed that in step one, teaching program contributed significantly to the model, F(3, 124) = 8.27, *p* < 0.01, and explained 16.68% of the variation in autonomy score, while adding experience, education, and group size to the model did not result in a significant increase in R^2^ (Table 4). The hierarchical multiple regression for the subscores of competence and relatedness yielded no significant models, with no or very little explanatory value of the predictors (see Table 4).

**Table 4:** Hierarchical multiple regression for total SDT score, and autonomy, competence, and relatedness subscales.

	Total SDT score				Autonomy				Competence				Relatedness			
Predictor	*R* ^2^	*R*^2^ change	F	*p*	*R* ^2^	*R*^2^ change	F	*p*	*R* ^2^	*R*^2^ change	F	*p*	*R* ^2^	*R*^2^ change	F	*p*
Model 1																
1. Teaching program	0.11	0.11	4.87	<0.01	0.17	0.17	8.27	<0.01	0.05	0.05	2.29	0.08	0.01	0.01	0.35	0.79
Model 2																
2. Experience	0.13	0.02	3.20	0.08	0.19	0.02	3.12	0.08	0.08	0.02	3.24	0.07	0.01	0.00	0.31	0.58
Model 3																
3. Education	0.18	0.05	1.18	0.32	0.25	0.06	1.47	0.20	0.13	0.05	1.06	0.39	0.04	0.03	0.59	0.74
Model 4																
4. Group size	0.18	0.00	0.08	0.78	0.25	0.00	0.20	0.65	0.13	0.00	0.11	0.74	0.04	0.00	0.45	0.50

*R*^2^, explained variance; *R*^2^ change, change in explained variance; F, F statistic; *p*, *p*-value.

## 4. Discussion

The present study had three aims: (i) to examine to what extent Dutch swimming instructors cater to the three basic psychological needs during swimming lessons; (ii) to examine to what extent a teaching program based on SDT prompts swimming instructors to better meet the three basic psychological needs of SDT, and (iii) to examine to what extent the teaching program, the instructor's education and experience, and the group size predict the employment of SDT in swimming lessons by swimming instructors.

Before discussing the findings in light of our expectations and previous research findings, we address the SDT teaching style scale's validity and reliability. Previous studies already confirmed the SDT teaching style scale's internal validity and reliability (16, 23). The present study aimed to replicate these findings and to expand them by seeking to confirm the scale's structural validity.

First, regarding the scale's structural validity, when the original SDT teaching style scale was analyzed with the inclusion of item R3 (interaction between children), it yielded somewhat contradictory results. Some fit indices indicated acceptable to good levels of fit, while a few other fit indices had a poor fit. The model's fit indices were significantly enhanced by eliminating item R3 from the scale. We reasoned that promoting interactions between children would be an important manifestation of relatedness supporting behavior (46). This item however did not correlate with the interaction between the instructor and the children, suggesting that behaviors potentially strengthening the interaction between instructor and the children and interaction among children are independent aspects of relatedness. Fedesco, Bonem, Wang, and Henares (47) found similar results, highlighting the multidimensional nature of relatedness support. Altogether, we decided to eliminate item R3. Following this elimination, all fit indices became good to excellent confirming the scale's validity in distinguishing autonomy, competence, and (child-instructor) relatedness from one another.

The scale's interrater reliability was good to excellent. The internal consistency was acceptable for the subscales autonomy and relatedness, but poor for the unmodified subscale competence. Especially item C1 (degree of differentiation) displayed a lack of coherence with the remaining items on the competence subscale. It was reasoned that more differentiation would support perceived competence (23, 48) as instructors would provide assignments based on children's swimming level. Alternatively, however, it cannot be ruled out that instructors refrained from differentiation during their lessons because of concerns that less competent children would feel disappointed when their peers were allowed to perform more challenging tasks (49). Despite this concern, we chose to retain item C1, because it was present in the original scale of Tessier et al. (23), which demonstrated good internal consistency.

The scale's minimal detectable change was 0.16 points at the group level and 1.11 points at the individual level, suggesting that the scale is suitable to compare the employment of SDT across different groups, such as teaching programs, but less suitable for monitoring changes in the employment of SDT by individual instructors.

Altogether, the scale's validity was confirmed for autonomy, competence, and relatedness. Moreover, the scale's internal consistency was corroborated for autonomy and relatedness. However, conflicting results were found for competence demonstrating poor internal consistency in our study, but good internal consistency in the study of Tessier et al. (23). Consequently, some caution should be exercised in interpreting the results on this subscale and further research on this issue is necessary.

Now that the SDT teaching style scale's validity and reliability have been assessed, we can proceed to discuss our main findings. First and foremost, Dutch swimming instructors scored on average 3.50, 4.33, and 5.50 points for the employment of autonomy, competence, and relatedness in swimming lessons, respectively. We found that the median subscore for autonomy was significantly lower than 4, while the median subscores for competence and relatedness were significantly higher than 4. This indicates that Dutch swimming instructors mostly showed autonomy-thwarting behaviors, competence-supportive behaviors, and relatedness-supportive behaviors during their swimming lessons. This result only partially supports our hypothesis that instructors would typically exhibit need-thwarting behaviors since this does not apply to competence and relatedness. However, the median score of 4.33 for competence suggests only weakly supportive behaviors, considering that the score is in close proximity to the threshold of 4 and is still significantly lower than the maximum score of 7 points. Moreover, the median score of 5.50 for relatedness is also still considerably lower than 7. Therefore, although there may be some incoherence between the behaviors on the competence subscale, these findings suggest there is still room to better cater to all three fundamental needs of SDT in swimming lessons in the Netherlands.

The present results are consistent with those of Reeve, Jang et al. (16), who studied the provision of autonomy, competence, and relatedness by high school teachers before and after a SDT intervention. Before the intervention, the teachers predominantly employed autonomy-thwarting, competence-supportive, and relatedness-supportive behaviors, as in our study. A deviant result in this regard was found by Jang, Reeve, and Deci (50), who observed that high school teachers in the United States of America mostly exhibited autonomy-supportive behaviors as indicated by a mean score of 4.59 points for autonomy. Several methodological aspects might account for these mixed findings, including differences in the SDT teaching style scales and procedures, and the small sample sizes used in most studies (16, 50).

Our observations show that Dutch swimming instructors cater significantly less to autonomy than competence and relatedness, with the latter being catered to most effectively. This may have several reasons. First, considering that there is always a risk of drowning while learning to swim, providing autonomy support during swimming lessons may endanger children's safety, which might prompt instructors to adopt a more controlling teaching style. Second, children in swimming lessons may be too young to respond positively to autonomy, or instructors may assume this to be the case, resulting in more controlling behaviors. Third, it might be that instructors are not familiar with the potential benefits of an autonomy-oriented teaching style, either because it was no part of their education or what they have been taught was not sufficiently applied in practice. As a result, instructors may believe that a controlling teaching style is necessary to teach children how to swim. However, it might well be that teaching children more playfully with a great deal of autonomy may result in the same or perhaps even better learning outcomes (15).

In fact, and this speaks to the study's second aim, our results indicate that it is feasible to enhance swimming instructors’ ability to cater to the need for autonomy through training, given that instructors affiliated with Easyswim succeeded significantly better in this regard than instructors following other teaching programs, not based on SDT. Moreover, it also seems feasible to encourage swimming instructors to strengthen children's perceptions of competence and relatedness during swimming lessons, as Easyswim instructors received higher scores in these areas, although the differences were not statistically significant. Roughly speaking, our findings are consistent with our expectation that SDT-based teaching would prompt swimming instructors to better cater to all three basic psychological needs.

The lack of statistical significance for competence and relatedness can be explained as follows. It could well be more difficult to improve swimming instructors' competence- and relatedness-supportive behaviors, as these two needs are already better implemented in swimming lessons than autonomy. This assumption is supported by the findings of Reeve, Jang et al. (16), who investigated whether high school teachers can be stimulated to expand their teaching styles to be more autonomy-, competence-, and relatedness-supportive by educating them about SDT. As the teachers mainly showed autonomy-thwarting, and competence- and relatedness-supportive behaviors before the intervention, the intervention predominantly enhanced teachers' autonomy support. Nonetheless, in later work, Cheon et al. (51) and Tessier et al. (23) showed that the catering of all three needs of the SDT—autonomy, competence, and relatedness—can be improved in class by providing systematic guidance to teachers on how to implement SDT principles in their lessons.

As regards the study's third aim, only the teaching program was found to be a significant predictor of the employment of SDT by swimming instructors. Instructor's education and experience, and the size of the swimming class, had no significant influence on the employment of SDT in swimming lessons. This result stands in contrast with our hypothesis and with previous research in educational settings showing that instructors' experience and group size did influence the employment of SDT in class (36, 37). Based on the present study, these findings can thus not be generalized to swimming lessons. Concerning instructor's education, the non-significant relationship may be caused because in many education programs, SDT is not addressed and if it is (as in general PE) then it is mostly in theoretical terms and not in how to integrate it in teaching. Consequently, more attention should be paid to SDT during and after an instructor's education. Regarding instructor's experience, Leroy et al. (36) stated that previous teaching experiences would support teachers to extend their teaching styles to become more autonomy-oriented. This may not be true for swimming because of the safety component present in swimming lessons. Lastly, the group size in swimming lessons tends to be much smaller compared to the group size in educational classes. This creates a different context, wherein the impact of group size on instructor's employment of SDT may no longer be a significant predictor. The unique dynamics and individual attention afforded by smaller groups may influence the instructor's approach to catering to students' psychological needs, rendering the influence of group size less prominent in this specific domain.

In conclusion it is useful to discuss the strengths and limitations of our study, beginning with the study's strengths. This research extends previous research findings obtained in educational settings demonstrating that is possible to expand a teacher's teaching style to become more autonomy-, competence-, and relatedness-supportive (22, 23). Our results showed that these results are generalizable to learning environments in which it is important to secure the learner's safety, such as swimming lessons, especially with respect to autonomy.

We established those results in a study with a considerably larger sample size than in previous research. With over 120 observations of swimming lessons provided by equally many instructors, the study involved a diverse group of instructors, providing an accurate and comprehensive assessment of the employment of the SDT in swimming lessons in the Netherlands. By including a large sample of instructors, working all over the Netherlands, our findings can be considered representative for the state of swimming education in the Netherlands, which may well be found to be representative for other Western countries as well. However, it should be explicitly noted that our study did not include the observation of swimming lessons in countries with different cultural, social, or economic backgrounds, which implies that the present findings should not be generalized to those countries in the absence of cross-cultural research on this matter.

As regard the study's limitations, one potential shortcoming is that we only observed relatedness supportive behaviors involving child-instructor interactions. Because of the exclusion of item R3 (interaction between children), no behaviors involving child-child interactions were observed. Consequently, no conclusions can be made about the relatedness among children during swimming lessons.

Another potential drawback could be that, although we asked the instructors to provide a swimming lesson as usual and did not inform them about the real aims of the observational study, the possibility remains that instructors unconsciously altered their teaching behaviors and made efforts to provide a more engaging and enjoyable lesson, given that their performance was being evaluated. Nonetheless, due to the large number of lessons observed, involving a diverse group of instructors, we believe these unconscious modifications in instructional behavior had little to no impact on our findings.

Another limitation is that we did not assess other factors that might have influenced the extent to which instructors employ the SDT in swimming lessons, such as personality traits (22) and the expectations of parents or supervisors (52). To examine those potential influences, an even more elaborate study would have been required than the present one, which was already quite large.

Since the insights we obtained can be applied to help improve swimming lessons for children, it is also useful to address their practical implications before formulating the study's general conclusion. For starters, the design of swimming lessons should facilitate a considerable level of autonomy-, competence- and relatedness support among children. This requires a markedly different orientation and skill set from instructors than currently utilized in more traditional teaching approaches that are predominantly focused on the improvement of swimming technique(s). Although it remains to be demonstrated, we believe that if teaching programs, and thus swimming instructors, would prioritize the development of a learning environment that offers abundant affordances and challenges for children, while simultaneously guiding and monitoring children's progress in swimming performance, children will not only develop their swimming skills but also their intrinsic motivation for swimming. To achieve this, swimming instructor's education programs should focus more on the development of pedagogical and didactic skills of (future) instructors, with specific emphasis on employing SDT in swimming lessons. At present, such an approach is lacking in most current education programs.

The findings for Easyswim demonstrate that it is possible to incorporate SDT into swimming lessons by employing its principles into a teaching program. However, this does not imply their teaching program is the best pedagogic or didactic teaching program, nor that this teaching program will result in the highest intrinsic motivation. Our findings do also not demonstrate that the teaching program of Easyswim will lead to better learning results and better swimming performance, nor to more children that keep swimming or join a swimming club. Further research is needed to investigate these aspects.

In conclusion, Dutch swimming instructors mostly exhibit autonomy-thwarting, weakly competence-supportive, and relatedness-supportive behaviors to satisfy SDT's three basic psychological needs during swimming lessons. This suggests that the basic need for autonomy is implemented to a lesser extent in swimming lessons than competence and relatedness, with the latter being employed most effectively. The findings also show that it is possible for swimming instructors to improve their autonomy-supportive behaviors if the teaching program is grounded in the principles of SDT. Children's intrinsic motivation to continue swimming beyond the completion of their teaching program(s) will likely benefit from such programs, potentially resulting in more children joining a swimming club and the maintenance of swimming skills over years.

## Data Availability

The raw data supporting the conclusions of this article will be made available by the authors, without undue reservation.
