# Supplementary material for: How intrinsically motivating are swimming instructors/lessons in the Netherlands? An observational study through the lens of self-determination theory
Source: Front Sports Act Living. 2023 Sep 20;5:1236256. doi: 10.3389/fspor.2023.1236256 (PMC10548366; doi:10.3389/fspor.2023.1236256)
Supplement: Supplementary file 1 [file Datasheet1.pdf]

Supplementary Material

How intrinsically motivating are swimming instructors / lessons in the Netherlands? An observational study through the lens of self-determination theory

Carola Minkels[1,2,*], Peter J. Beek[1,2†], John van der Kamp[2†]

[1]InnoSportLab de Tongelreep, Eindhoven, Netherlands

[2]Department of Human Movement Sciences, Vrije Universiteit Amsterdam, Amsterdam Movement Sciences, Amsterdam, Netherlands

*** Correspondence:**

Carola Minkels

c.a.p.minkels@vu.nl

1 Supplementary Data

1.1 Supplementary Material I: Modified version of the SDT teaching style scale developed by Tessier et al (2010)

Teacher's Autonomy Support

<u>Type of motivation</u>

Relies on extrinsic		Nurture inner motivational
sources of motivation	1 2 3 **4** 5 6 7	resources
**Offers incentives, consequences, directives*		**Interest, enjoyment, sense of challenge*
**Makes assignments, seeks compliance*		**Creates opportunities for initiative*

<u>Type of language</u>

Controlling language	1 2 3 **4** 5 6 7	Informational language
**Pressuring, ego-involving*		**Informational, flexible*
**Should, must, have to, got to*		**provide options and choices*
**Neglects importance of requests*		**Identifies importance of requests*

<u>Organizational instructions</u>

**Use commands and directives, imposes everything*	1 2 3 **4** 5 6 7	**Give choices and options*

<u>Provide choices in</u>

- Which activities
- Task order
- Task difficulty
- Use of devices
- Collaboration with other children
- Feedback

<u>Rationales</u>

**Imposes rules and limits*	1 2 3 **4** 5 6 7	**Explains rules and limits*
Doesn't give rationales		*Gives rationales*

Teacher's Provision of Structure

<u>Instructions given to the whole class</u>

Task structure

**Same task for all students*	1 2 3 **4** 5 6 7	**Differentiated and challenging tasks*

<u>Interaction teacher - student</u>

Structure of the student's activity

**No feedback, vague goals, confusing, unclear*	1 2 3 **4** 5 6 7	**Contingent feedback, short term goals*

Encouragement

**No encouragement*	1 2 3 **4** 5 6 7	**Encourages students' effort and progress*

Teacher's Provision of Involvement

<u>During task instructions to the whole class</u>

**Cold, distant, offers strict feedback, sarcastic learning*	1 2 3 **4** 5 6 7	**Sympathetic, warm, humorous*

<u>Interaction teacher - student</u>

**Cold, distant, strict, sarcastic, rigid, inflexible, limits time*	1 2 3 **4** 5 6 7	**Sympathetic, warm, uses humor with each student, listens understand students, invest time and energy*

<u>Interaction between children</u>

**No interaction between students, they may not practice together*	1 2 3 **4** 5 6 7	**Strong interaction between children often practice together*

1.2 Supplementary Material II: Questionnaire to obtain background information about the swimming lesson, the swimming instructor, and the swimming lesson school

1. What is the date?

2. What is your name?

3. What is your e-mail address?

4. What is your gender?

o Male

o Female

o Other

5. What is your date of birth?

6. How many years of experience do you have as a swimming instructor?

7. How many hours per week do you provide swimming lessons?

8. What specific swimming instructor education did you follow to be allowed to provide swimming lessons?

9. What is the name of your swimming school?

10. Which teaching program does this swimming school offer?

o Easyswim

o ZwemABC

o Optisport

o SuperSpetters

o Other

11. Which swimming certificate does this swimming school issue?

o Easyswim

o ZwemABC

o Optisport

o SuperSpetters

o Other

12. How long does it take to complete the entire swimming program and achieve your swimming certificate?

13. Under which organizational structure does the swimming school operate?

o Municipality

o Swimming school

o Swimming club

o Other

14. What is the swimming level of the children participating in the swimming lesson?

15. How long lasts the swimming lesson?

16. How many times a week does this group follow swimming lessons?

17. How many swimming instructors provide the swimming lesson?

18. How many children are in this swimming lesson?

19. What is the age range of the children in this swimming class?

1.3 Supplementary Material III: Swimming instructors’ educations accepted by the NRZ.

Accepted swimming instructors’ educations by the NRZ

- Aqua Zuyd Opleidingen
- Endurance
- Fellinco
- Gemeente Amsterdam
- Gemeente Oosterhout / Recreatiecentrum de Warande
- HeBe Training en Advies
- HR Opleiders
- JoBa opleidingen
- Let us train you
- Linda Berg Trainingen & Opleidingen
- Nationale Beroepsopleiding Zwemonderwijs (NBZ)
- Nederlandse Culturele Sportbond
- Opleidingscentrum Sportbedrijf Tilburg
- Propulz.tP
- Sabrina Traint
- SOZON
- Sport Opleiding Nederland
- Swimpy
- SwimTechNL
- Training Centrum Emmen
- Van Kuijen Educatie
- Veldkamp Zwem Opleidingen
- Walderveenbad, Loosdrecht
- ZwemNetwerk
- Zwemschool de Zwaantjes
- HBO ALO
- MBO sport & bewegen
- Zweminstructeur niveau 3 KNZB
- ProOzo Professioneel Zweminstituut Zwemonderwijs
- Certificeerbare eenheid ‘Lesgever ZwemABC’ van Allround Zwembadmedewerker
- Optisport opleiding tot zweminstructeur
- ENVOZ-zwemonderwijzer
- Aqua Leisure Host niveau 3
- Sportfondsen

1.4 Supplementary Material IV: Swimming instructors' educations included for analysis

Swimming instructors' educations included for analysis

Education	n
General PE teacher education	46
General swimming instructor education	8
Swimming instructor education tailored to a specific program (Easyswim)	6
Swimming instructor education tailored to a specific program (ZwemABC)	28
Swimming instructor education tailored to a specific program (SuperSpetters)	22
Undiplomatized	10
Other	8
